# Immediately provisionalized tapered conical connection implants for single-tooth restorations in the maxillary esthetic zone: a 5-year prospective single-cohort multicenter analysis

**DOI:** 10.1007/s00784-021-04328-2

**Published:** 2022-01-08

**Authors:** Russell A. Baer, Robert Nölken, Snjezana Colic, Guido Heydecke, Christine Mirzakhanian, Alexandra Behneke, Nikolaus Behneke, Edward Gottesman, Liliana Ottria, Alessandro Pozzi, Alexander Fügl, Werner Zechner

**Affiliations:** 1 University Associates in Dentistry, Chicago, IL USA; 2 University Medical Center of Johannes Gutenberg University Mainz, Mainz, Germany; 3 Private Practice, Lindau, Germany; 4 University of Belgrade, Belgrade, Serbia; 5 University Medical Center Hamburg-Eppendorf, Hamburg, Germany; 6 Private Practice, New York, NY USA; 7 University of Rome Tor Vergata, Rome, Italy; 8 Dental University Clinic, Medical University of Vienna, Vienna, Austria

**Keywords:** Conical connection, Immediate provisionalization, Anterior maxilla, Soft tissue response, Bone remodeling, Esthetics

## Abstract

**Objectives:**

This open, single-cohort, multicenter, prospective study investigated the efficacy of immediately provisionalized tapered conical connection implant for single-tooth restorations in the anterior and premolar regions of the maxilla after 5 years of function.

**Materials and methods:**

All implants were placed in healed sites and immediately provisionalized. MBLs, soft-tissue parameters, and oral-health impact profile (OHIP) were evaluated at implant insertion, 6, 12, 24, 36, and 60 months. Paired Wilcoxon signed-rank tests and Kaplan–Meier survival analysis was used for statistical and implant survival/success analyses, respectively.

**Results:**

Seventy-seven patients (81 implants) completed the 5-year follow-up. The 5-year cumulative survival and success rates were 97.8%, and the mean MBL change from implant insertion to 5 years was − 0.80 ± 1.13 mm. Optimal papilla index scores were observed at 90.1% of sites at 5 years compared with 32.8% of sites at insertion. Pink esthetic score, modified bleeding and plaque indices, and OHIP showed statistically significant improvement at the 5-year follow-up.

**Conclusions:**

Immediately provisionalized tapered conical connection implants promote marginal bone stability and excellent esthetic outcomes after 5 years of function.

**Clinical relevance:**

This treatment is a viable option for patients requiring immediately provisionalized single-tooth restorations in the esthetic zone and shows favorable long-term clinical outcomes, including marginal bone stability and excellent esthetics.

## Introduction

The goal of modern implant dentistry is to not only achieve implant survival but also to ensure an esthetic and functional restoration that is compatible with the existing dentition. This is particularly relevant for the anterior maxilla, where the teeth and surrounding structures are clearly visible and, therefore, have a direct impact on the patient’s quality of life. Single-tooth implant placements in the esthetic zone have become a reliable treatment option, with high implant survival rates [1]. Therefore, the focus has now shifted toward achieving optimal hard- and soft tissue responses, both immediately after placement as well as in the long term, to obtain favorable esthetic and patient-centered outcomes [2, 3]. Besides stable and healthy peri-implant tissues, other variables including, but not limited to, the patient’s smile line, tooth position and morphology, root position of the adjacent teeth, periodontium biotype, bone anatomy at the implant site, and optimal implant positioning, can also influence the predictability of esthetic outcomes [4].

Current literature suggests that restoring single missing teeth in the esthetic zone by placing implants into fresh extraction sockets may be advantageous compared with placing implants into healed sites because this approach often reflects patient wishes, and it prevents ridge resorption following tooth loss [5]. However, immediate placement is not always possible if the patients have congenitally missing teeth, if they had lost teeth as children and were thus not eligible for implant therapy, or were not able to receive implants for other reasons. Placing implants into healed sites in the esthetic zone of the maxilla is associated with several challenges. The loss of bone volume due to ridge resorption requires careful planning to allow for optimal implant position, and the surgical technique must anticipate the planned soft tissue margin to recreate natural-looking esthetics.

Good long-term functional and esthetic outcomes of implant-supported tooth restorations hinge on the successful osseointegration of the implant to promote healthy and stable marginal bone and peri-implant soft tissues. A tapered implant geometry facilitates placement into tight spaces [6] and allows the gradual expansion of the alveolar bone ridge, minimizing stress to the surrounding bone [7] and promoting primary stability. Conical implant-abutment connections alleviate mechanical strain on the crestal bone, reducing micromovements and marginal bone loss [8–11], and facilitate platform shifting, allowing for biological width expansion, reducing crestal bone loss, and improving the stability and maintenance of peri-implant soft tissue in the esthetic zone [10, 12, 13]. Platform shifting of the implant-abutment junction inwards also relocates possible bacterial growth and inflammatory infiltrates away from the crestal bone, reducing crestal bone resorption [14–16].

This open, single-cohort, prospective, multicenter study aimed to assess the long-term (5-year) survival and esthetic outcomes following immediate provisionalization of the NobelReplace Conical Connection (NRCC) implant (Nobel Biocare AB, Göteborg, Sweden), a tapered implant that uses an internal hexagonal interlocking conical connection and platform shifting, into healed anterior and premolar maxillary sites. The primary objective of this study was to evaluate the change in peri-implant MBLs. Secondary objectives included implant success and survival, soft tissue response, esthetic outcomes, and oral health-related quality of life. The 1- and 3-year esthetic and functional outcomes of this study have been previously published [17, 18]. This manuscript describes the 5-year final results.

## Materials and methods

The study parameters and methods have been previously described in detail [17, 18]. Briefly, this open, single-cohort, multicenter, prospective study enrolled patients (18 years of age or older) requiring single-tooth restorations in the anterior and premolar regions of the maxilla (Fédération Dentaire Internationale (FDI) numbering, 15 − 25) between March 21, 2011, and July 5, 2013, at one of the eight participating private practice clinics and academic hospital-based institutions located in Austria, Germany, Italy, Serbia, and the USA. Centers were selected according to prior experience with the implant used in this study and clinical compliance. Approvals were obtained from the ethical oversight committees at each center (Center 1, Barlattani_Pozzo: REGISTRO SPERIMENTAZIONI 56/11, 23 May 2011; Center 2, Baer: IRB 11,041–01, 1 Mar 2011; Center 3, Behneke, 837.214.11(7456)_Landesärztekammer Rheinland-Pfalz 06 Sep 2011; Center 4, Heydecke, PV3756_EK der Ärztekammer Hamburg, 25 May 2011; Center 5, Gottesman, IRB 11,041-0A1, 15 Aug 2011; Center 6, Nölken, 01/1203_Freiburger EK International; Center 7, Zechner, EK Universität Wien, EK Nr. 356/2011 10 May 2011; Center 8, Colic, 36/12_Stomfak, 09 Jul 2012). This study is reported in accordance with the Strengthening the Reporting of Observational Studies in Epidemiology (STROBE) statement [19].

Based on the study’s inclusion and exclusion criteria [17, 18], patients were selected and rehabilitated using single tooth restorations supported by NRCC implants placed in healed sites. All implants were placed by experienced surgeons who received training on the study protocol prior to the start of the trial. The insertion torque used was 35–45 Ncm, as measured using a manual torque wrench; however, due to the inaccuracy of the wrench, torque values between 30 and 50 Ncm were allowed. Implant stability was tested manually by tapping or rocking the implant with a hand instrument, and further assessments were performed at the clinician’s discretion. Bone quality and quantity were assessed at the time of surgery, according to the Lekholm and Zarb classification. The need for bone or soft-tissue grafting and the choice of grafting technique was determined on a case-by-case basis by the treating clinician at the time of implant placement. All implants were provisionalized within 24 h following the surgery through the placement of a cement- or screw-retained provisional crown on a temporary titanium abutment. Patients received the definitive prosthesis, a cement- or screw-retained NobelProcera crown with a titanium or zirconia abutment within 6 months after implant placement. The choice of abutment and retention type was left to the discretion of the treating clinician to ensure they met the individual patient’s needs.

Study outcomes included MBL changes, implant survival, implant success, esthetic parameters such as papilla index, Mombelli’s modified bleeding index, plaque accumulation, and PES, and oral health-related quality of life. All outcomes were assessed at six time points–baseline (implant insertion) and 6, 12, 24, 36, and 60 months postinsertion. MBLs were measured as the distance between the most apical bone level and the implant-abutment junction, based on standardized intraoral periapical radiographs acquired using individual bite blocks. The distance was then calibrated based on the implant diameter. Marginal bone remodeling (MBL*∆*) was calculated as the difference in bone levels between baseline (implant insertion) and each follow-up time point, with negative values indicating bone loss. The papilla health adjacent to the implant was assessed using Jemt’s papilla index [20]. Sulcus bleeding and plaque accumulation were evaluated using a modified sulcus bleeding index and plaque index, respectively [21]. PES measurements were conducted as described by Furhauser et al. [22]. An additional PES analysis was performed according to Hof et al. [23] to observe the changes in the frequency of “unsatisfactory” scores (overall PES ranked from 0 to 9) and “satisfactory” scores (overall PES ranked from 10 to 14) over time. At each follow-up visit, the OHIP of the patients was evaluated using the OHIP-14 questionnaire, which primarily aimed to gauge the improvements in the patient’s quality of life as a result of implant treatment. With four answer choices to assess the frequency of functional and esthetic complaints (0: never; 1: hardly ever; 2: fairly often; 4: very often) provided for a total of 14 questions, the OHIP-14 score was calculated by adding up the scores for all the questions for every patient and averaged per time point, with the lower score demonstrating the better oral health-related quality of life.

A single examiner evaluated PES (Dr. Strbac), and a second examiner measured bone levels on radiographic images (Dr. Lith). Due to the broad experience of both of these examiners, no evaluations were performed by additional examiners, and thus inter-rater variability was not assessed. The intra-rater reliability scores were 96.4% for PES and 88.7% (within 0.5 mm) for radiographs. All radiographs, clinical pictures, and other study data were stored in the electronic database Viedoc 3ä, electronic data capture system provided by Pharma Consulting Group 2004–2016 (Kungsängsvägen 19, S-753 23 Uppsala, Sweden).

Implant survival was assessed based on whether the implant remained functional at each time point. Implant success was evaluated based on the success criteria outlined by van Steenberghe, and a successful implant was defined as one that (1) does not cause allergic, toxic, or gross infectious reactions either locally or systemically; (2) offers anchorage to a functional prosthesis; (3) does not show signs of fracture or bending; (4) does not show any mobility when individually tested by tapping or rocking with a hand instrument, or when tested with an electronic tapping device does not reach improper values of rigidity; (5) does not show any signs of radiolucency on an intra-oral radiograph using a paralleling technique strictly perpendicular to the implant-bone interface [24].

### Statistical analysis

Sample size calculation was performed for a single-arm study with a 5-year mean marginal bone remodeling rate (MBL*∆*) compared to the weighted mean MBL*∆* of − 0.78 mm observed at 1-year post-implant insertion in 2 other studies using tapered design implants with an internal tri-channel connection (NobelReplace Tapered Groovy)[25, 26]. Based on the test for non-inferiority, using a two-sided, inclusive 95% confidence interval, a significance level of *α* = 0.05, a power of 80%, and compensation for 20% subject withdrawal, a total of 96 subjects were included. The statistical evaluation considered all collected data from surgery and follow-up procedures. Missing data were not imputed or included in the statistical evaluation. The distribution of continuous variables is reported as the mean and standard deviation (SD), whereas categorical variables are reported as the frequency and percentage. The cumulative survival rate (CSR) and success rate of implants were assessed using Kaplan–Meier analyses. Unsuccessful implants that eventually recovered during the course of the study and withdrawn patients/implants were not included in this analysis. Changes in the papilla, Mombelli’s modified bleeding index, and plaque index between the different FUP time points were analyzed using paired Wilcoxon signed-rank tests. A *p*-value < 0.05 was considered significant. To investigate the possible factors associated with marginal bone loss, a generalized estimating equation (GEE) Gaussian model was employed, using overall PES, modified bleeding index, modified plaque index, Jemt’s papilla index, and overall OHIP-14 score as independent predicting variables. All statistical analyses were performed using SPSS Statistics version 25 (SPSS Inc., Chicago, IL, USA), with the exception of the GEE model, which was evaluated using R3.6.1 [27].

## Results

### Baseline characteristics

This study initially enrolled 101 patients requiring single-tooth restorations in the anterior or premolar maxilla. Of those, 7 patients (7 implants) were not considered eligible for the study after source data verification revealed that they failed to meet the inclusion/exclusion criteria. In total, 94 patients received 99 implants, with 5 patients treated with two implants each. Females comprised 57.4% of the study participants, and the average patient age at the time of implant placement was 41.1 ± 14.3 (mean ± standard deviation, SD) years. A majority (85.1%) of study participants were nonsmokers. Medical history records, which were available for all 94 patients, showed most of them to be in good overall health, with only 4 and 3 study patients reporting previous or ongoing illness, respectively. Two (2.1%) patients had a history of non-severe bruxism and four (4.2%) of periodontitis.

A total of 99 NRCC implants were placed in the extended esthetic zone of the maxilla, including the anterior and premolar regions. Of those, 57 implants (57.6%) had a narrow platform diameter (3.5 mm), and the remaining had a regular platform diameter (4.3 mm). Implants were placed either in healed sites (89.9%) or extraction sites that had healed for at least 8 weeks (10.1%). With regard to bone quantity, 94 sites (94.9%) showed little or moderate resorption, while the remaining 5 sites had advanced residual ridge resorption, including 1 site also showing some resorption of the basal bone. The distribution of hard vs. soft bone was comparable, with 53 sites (53.5%) classified to the former category. Most implants were inserted using a flap approach with (23.2%) or without (72.7%) releasing incisions, and 4 implants (4.0%) were placed using a flapless procedure. Soft tissue grafting, using connective tissue grafts, was used for 13 implant placements. Five implant sites had received bone graft material prior to the start of the study, and concomitant bone grafting was performed during 16 implant placements. Implants were placed using an average insertion torque of 39.3 ± 5.0 Ncm (range, 30–50 Ncm). The mean insertion torque in the soft bone (*n* = 46) was 38.1 ± 4.5 Ncm (range, 30–45 Ncm), which was only slightly lower than that of 40.1 ± 5.2 Ncm (range, 30–50 Ncm) recorded at implants placed in the hard bone (*n* = 53). Manual stability testing showed that 100% of the implants were stable at the time of insertion.

At insertion, 86 implants received temporary abutments, and the remaining 13 implants received final abutments. Both screw (43.4%) and cement retention (56.6%) were used. A total of 96 implants in 91 patients received the definitive prosthesis: zirconia and titanium abutments were used for 57 and 22 implants, respectively, and the remaining 17 implants received the esthetic abutment. With regard to the final prosthesis, which on average took place was 4 ± 1.7 months post-implant insertion (range, 0–13.6 months), 78 implants (81.3%) received a Nobel Procera crown, and 18 implants (18.8%) received other types of prosthesis, such as porcelain fused to metal or ceramic screw-retained crowns.

### Post-implantation follow-up

**Fig. 1:**
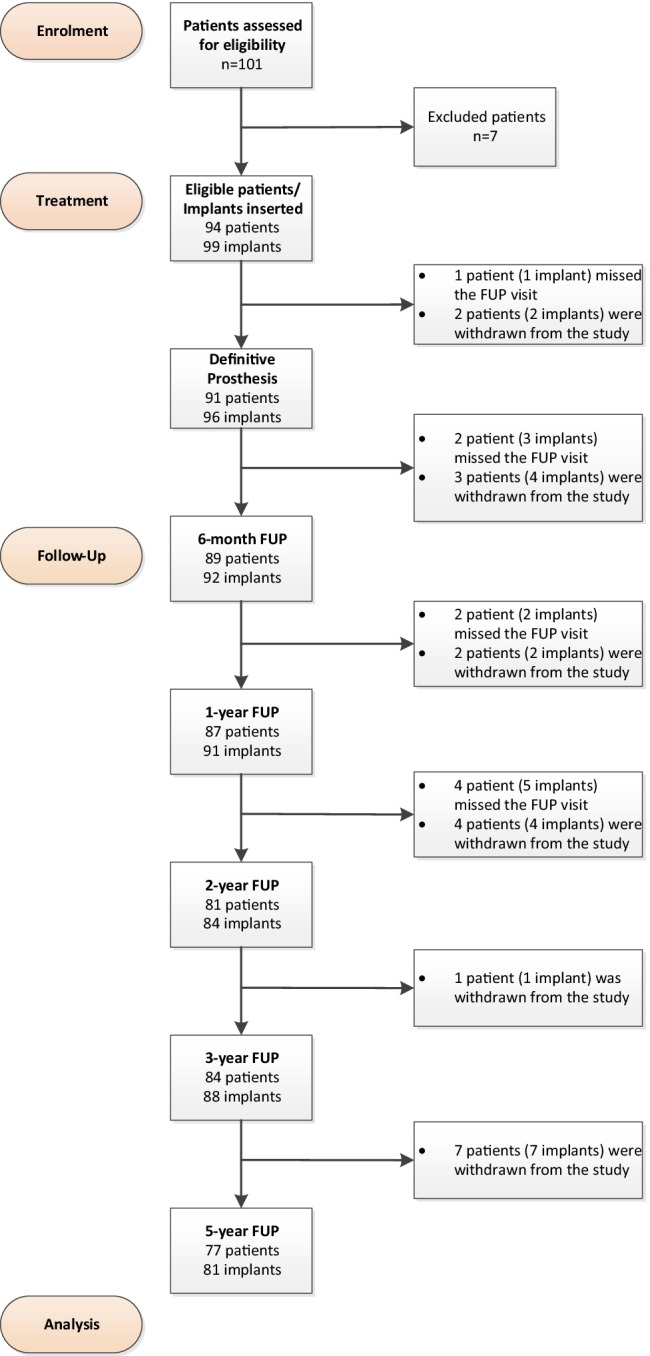
Study flow diagram from enrolment to final follow-up 5 years post-implant insertion

Of the 94 patients with 99 implants treated at the start of the study, 77 patients with 81 implants completed the 5-year follow-up. Figure 1 provides the study flow diagram detailing the number of patients and implants assessed at each follow-up visit.

### Study outcome

MBLs were radiographically assessed at implant insertion and at each of the five follow-up time points. At implant insertion (baseline), the mean MBL was − 0.38 ± 0.74 mm (*n* = 96). After the expected remodeling the mean MBL decreased to − 1.33 ± 1.06 mm at 6 months post-insertion (*n* = 91), and remained stable thereafter, with the mean MBL of − 1.28 ± 1.13 mm at 1 year (*n* = 91), − 1.02 ± 0.73 mm at 2 years (*n* = 80), − 1.14 ± 1.00 nm at 3 years (*n* = 84), and − 1.19 ± 0.95 mm at 5 years (*n* = 74). Calculation of the MBL changes reflected these values, with the statistical comparison revealing that only the MBL loss from implant insertion to the 6-month follow-up was statistically significant (Table 1). The primary objective of this study was to compare of the change in peri-implant MBLs, which was − 0.80 ± 1.13 mm (i.e., bone loss) from implant insertion to 5 years (*n* = 71), to the weighted MBL change of − 0.78 mm (bone loss) based on the reference group studies from two other studies using an implant with an identical geometry but an older, tri-channel implant-abutment connection [25, 26]. In a statistical test, the results from the current study were found to be non-inferior.

**Table 1:** Marginal bone level changes throughout the study. Negative values indicate bone loss

	Implant insertion to 6-month FUP	Implant insertion to 1-year FUP	Implant insertion to 2-year FUP	Implant insertion to 3-year FUP	Implant insertion to 5-year FUP	6-month to 1-year FUP	1- to 2-year FUP	1- to 5-year FUP	2- to 3-year FUP	2- to 5-year FUP	3- to 5-year FUP
Mean	− 0.92	− 0.86	− 0.61	− 0.74	− 0.80	0.05	0.22	0.07	− 0.08	− 0.14	− 0.08
Median	− 0.92	− 0.66	− 0.61	− 0.69	− 0.62	0.08	0.05	0.03	− 0.05	− 0.07	− 0.12
SD	1.24	1.35	0.95	1.20	1.13	0.75	1.04	1.06	0.79	0.73	0.75
Min	− 6.22	− 7.13	− 3.74	− 5.00	− 4.49	− 3.07	− 2.11	− 2.13	− 3.70	− 2.28	− 2.93
Max	1.80	3.07	1.52	1.79	1.07	3.15	5.84	4.27	1.92	1.48	2.12
N	89	88	78	81	71	88	78	74	77	67	70
*P*-value	** < **0.0001	** < **0.0001	** < **0.0001	** < **0.0001	** <** 0.0001	0.414	0.376	1.00	0.912	0.365	0.606

*FUP*, follow-up; *N*, number of implants; *SD*, standard deviation

A total of two implant failures occurred during the course of this study–one implant failed at 20 months post-insertion due to peri-implantitis, and the other failed at 52 months post-insertion due to mobility. The 5-year CSR calculated using the KM analysis was 97.8%. The details on the two failed implants are provided in Table 2.

**Table 2:** Detailed characteristics of the two implant failures recorded in the study

Implant #	Time of failure (since implant placement)	Patient characteristics				Implant/implant site characteristics					
		Gender	Age	Smoking status	Health status	FDI position	Augmentation	Implant size	Final torque	Bone quantity	Bone quality
Failed implant #1	20 months	Female	32	< 5 cigarettes per day	•Allergic to penicillin •No ongoing illness •No bruxism •No prior/ongoing periodontitis	25	Horizontal bone augmentation	4.3 × 11.5 mm	35 Ncm	B	1
Failed implant #2	52 months	Male	34	6–10 cigarettes per day	•No allergies •No ongoing illness •No bruxism •No prior/ongoing periodontitis	12	None	3.5 × 10 mm	35 Ncm	B	3

During the course of the study, 4 out of 99 implants had at some point shown signs of mobility and were, therefore, at that point reported as unsuccessful. In two of these cases, implants have shown asymptomatic mobility within the first year from placement, but the mobility was associated with no pain or swelling. In one patient, the crown was removed while the abutment was immobile within the implant, and the implant was loose. The loosening was visually recognizable, and the periotest value was + 9. The crown was not reinserted to avoid stress, and the abutment was left in situ because the implant was judged as too unstable. The implant became stable again over the following months, with continuously improving periotest values recorded at each follow-up visit; 6 months later, the periotest value was − 4, and tapping showed the implant was stable; thus, the crown was reinserted. In the other patient, implant mobility was also visible and confirmed using the periotest (value of + 12 was recorded) upon crown removal. The temporary crown was shortened occlusally to reduce the load and, during the next months, the implant regained stability. The definitive crown was placed 8 months later. In sum, applying the conservative approach to count the two implants that did recover as unsuccessful, the 5-year cumulative success rate was calculated to be 95.6%. Considering the two implants that recovered as successful yielded the 5-year cumulative success rate of 97.8%.

**Fig. 2:**
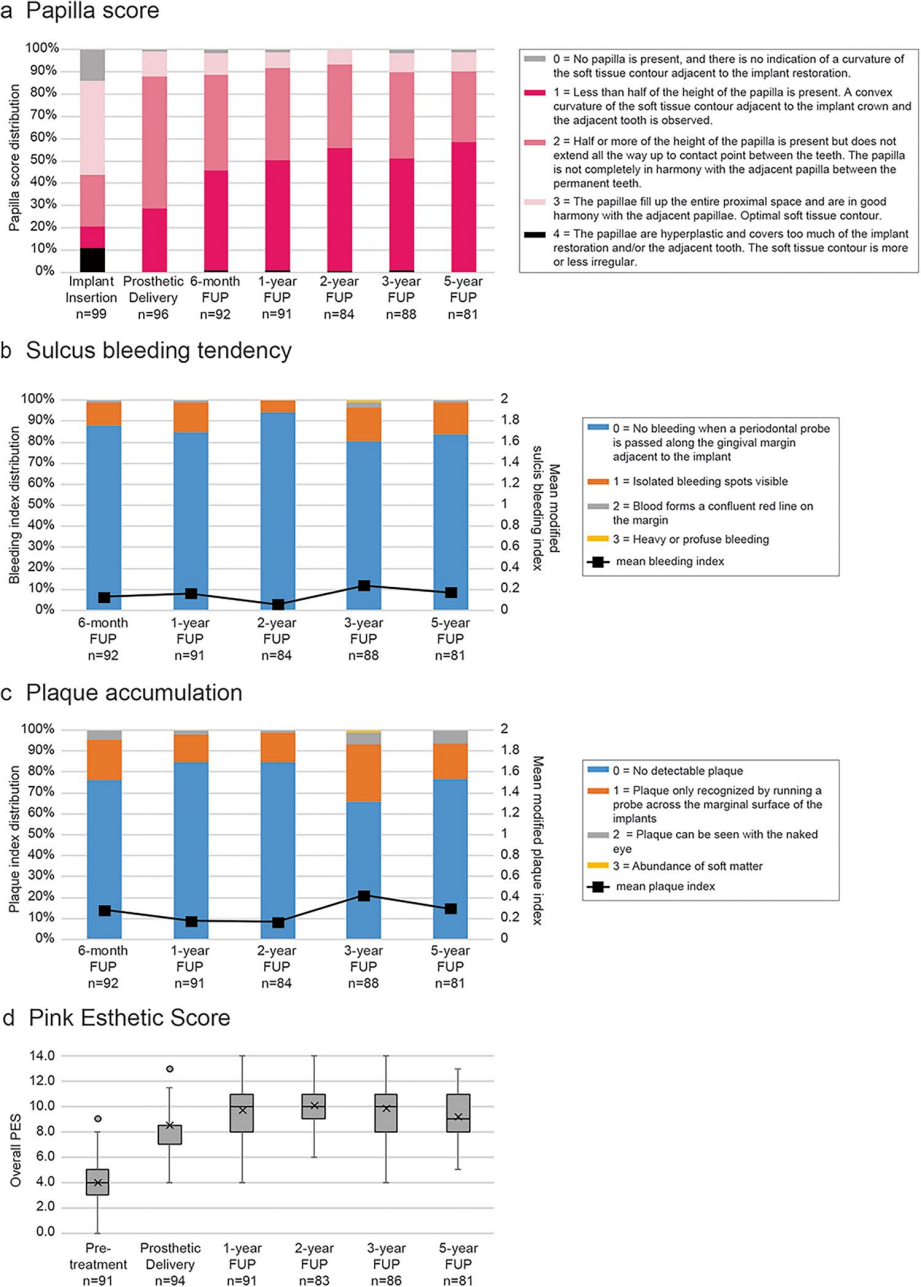
Soft tissue response throughout the study. Changes in the distribution of Jemt’s papilla score (**a**), sulcus bleeding index (**b**), plaque index (**c**), and PES (**d**). The number of assessed sites is listed for each FUP. The overall PES (**d**) is depicted in a box-and-whisker plot, with the means indicated as crosses and outliers as circles

Overall, peri-implant soft tissue health has improved significantly from implant insertion to prosthetic delivery and remained stable thereafter (Fig. 2 and Table 3).

**Table 3:**
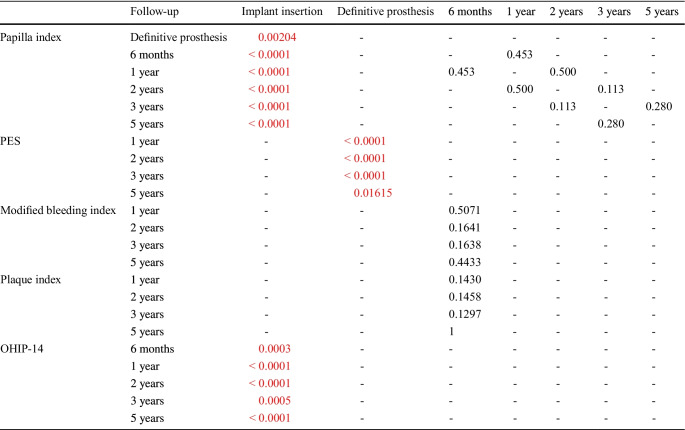
Statistical comparison of soft tissue health and QoL measures by follow-up. All significant p-values are listed in red

**Fig. 3:**
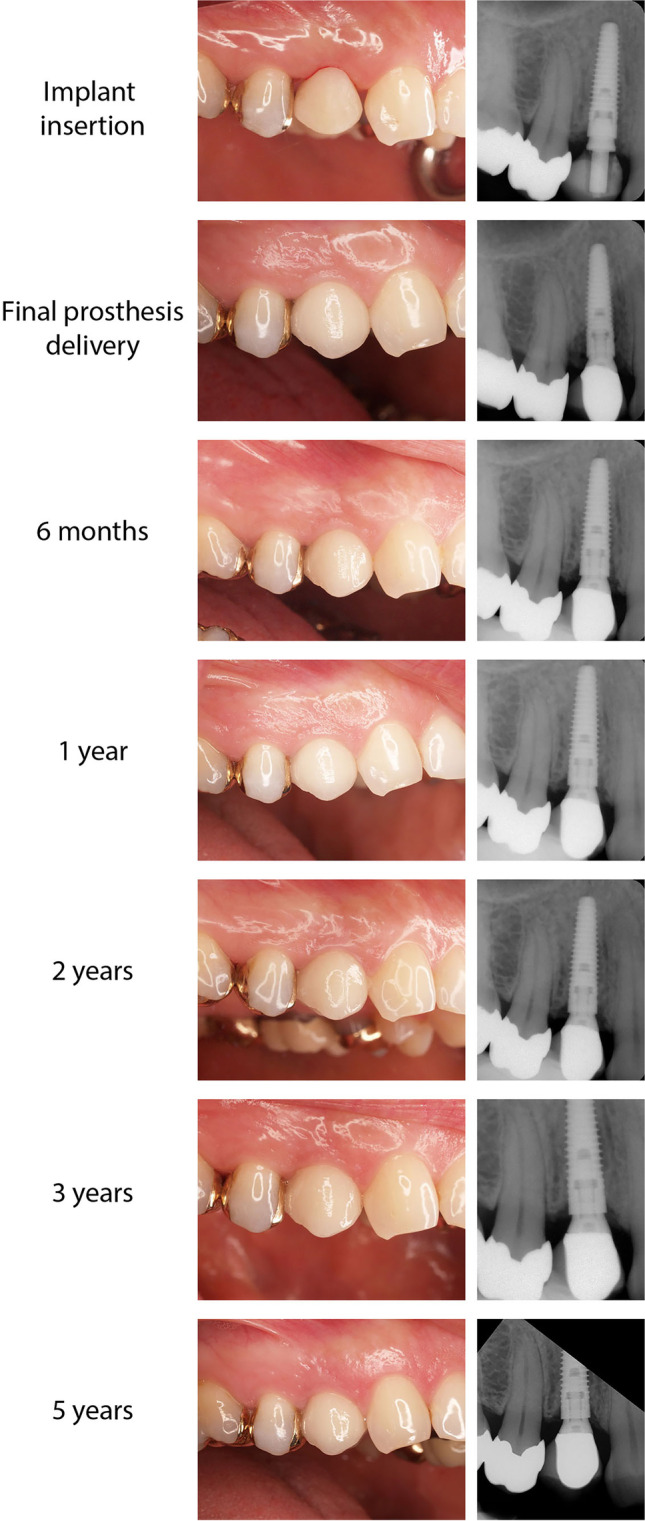
Representative clinical case. A 45-year-old female (nonsmoker) with no history of periodontitis or other parafunctional tendencies received a 13-mm long and 3.5-mm diameter NobelReplace CC implant to replace the missing first premolar tooth in the maxilla (FDI position 14). The bone at the insertion site was assessed as hard (quality 2), and the final insertion torque was 45 Ncm. The implant was immediately provisionalized, and the patient attended follow-up visits until study completion. Clinical images (left panels) and radiographs (right panels) acquired at indicated time points reveal healthy hard and soft tissue responses, accompanied by a visible improvement in esthetics

The papilla index score has improved significantly from implant insertion to all follow-up time points (all *p* < 0.05; Fig. 2a). At 5 years, 90.5% of the papilla received a papilla index score of 2 or 3. Favorable outcomes were also observed when evaluating Mombelli’s modified bleeding index, with 98.8% of sites showing no bleeding or only isolated bleeding spots at 5 years (Fig. 2b). Plaque accumulation was minimal, with 93.8% of sites showing no or little plaque at 5 years (Fig. 2c), while the PES evaluation revealed a statistically significant improvement from the time of definitive prosthesis delivery up to each follow-up time point (all *p* < 0.05, Fig. 2d). Furthermore, an additional PES analysis performed according to Hof et al. [23] showed that the frequency of satisfactory [10, 14] versus unsatisfactory (0 − 9) scores increased over time from 24.5% at definitive prosthesis delivery to 45.7% at the 5-year follow-up, with the frequency of scores peaking at 60.2% at the 2-year follow-up), in comparison with 100% of the implant sites showing unsatisfactory scores at the time of insertion. Figure 3 features a representative clinical case followed until the study end, 5 years post-implant insertion.

As expected, the high implant survival and success rates combined with the good peri-implant tissue health were reflected in continuing improvement in patient oral health-related quality of life. The decrease in functional and esthetic complaints was evident throughout the duration of the study, with the mean OHIP-14 score of 11.45 ± 9.65 at pretreatment (*n* = 94), 8.35 ± 9.14 at implant insertion (*n* = 94), 3.93 ± 6.08 at definitive prosthesis delivery (*n* = 91), 2.48 ± 5.43 at 6 months (*n* = 89), 1.62 ± 4.63 at 1 year (*n* = 87), 0.99 ± 2.72 at 2 years (*n* = 81), 1.46 ± 3.84 at 3 years (*n* = 84), and 0.84 ± 2.93 at 5 years (*n* = 77), with all follow-up values being significantly better than at implant insertion (all *p* < 0.001).

The analysis for factors that might influence marginal bone remodeling using the GEE model revealed no significant associations, i.e., none of the estimate coefficients were above 0.1 (Table 4). The model converged at the first iteration, furthermore, removal of a single predicting variable (OHIP-14) did not affect the estimate coefficients, suggesting model robustness.

**Table 4:** GEE modeling results to assess possible influence of soft tissue parameters, plaque accumulation, and OHIP-14 score on marginal bone loss

Independent variable	Estimate coefficient	Robust standard error
Overall PES	0.016	0.018
Modified bleeding index	0.068	0.046
Jemt’s papilla index	** − **0.021	0.053
Modified plaque index	0.086	0.040
Overall OHIP-14 score	0.001	0.003

### Complications

Complications and adverse events (AEs) were monitored during the entire study period. No serious AEs were reported at any point during the study; however, a total of 9 non-serious AEs were reported, including 4 that were device-related (2 were associated with implant mobility, 1 with pain, and 1 with peri-implantitis) and 5 that were not device-related.

## Discussion

This prospective multicenter study evaluated the 5-year functional and esthetic outcomes of immediately provisionalized, tapered conical connection implants with built-in platform shifting for single-tooth implant-supported restorations in the anterior and premolar regions of the maxilla.

The primary objective of this study was to evaluate the change in peri-implant MBLs and compare it to the MBL in reference group from two other studies using an implant with identical geometry but an older, tri-channel implant-abutment connection: statistical analysis revealed that the MBL changes observed in this study were non-inferior to the changes in the reference group, despite the fact that the MBL changes in the reference group were collected after only 1 year in function.

Marginal bone stability is a critical factor that determines implant success. Despite many efforts to prevent it, a certain amount of marginal bone loss inevitably occurs at the implant-neck level after implant insertion [28–31]. According to the implant success criteria in the context of bone remodeling proposed by Albrektsson [30], marginal bone losses of up to 1 mm are acceptable in the 1st year after implant loading, with subsequent annual losses of up to 0.2 mm. In this study, the mean marginal bone loss between baseline and the 1-year follow-up was 0.86 ± 1.35 mm, which fulfills the implant success criteria. Importantly, after the initial bone remodeling which reflects the adaptive response to the surgery and loading [32], the marginal bone levels remained stable throughout the study period. Very likely due to MBL stabilization, the study showed very good survival and success outcomes, with the 5-year CSR and cumulative success rates of both 97.8%. The success rate considers the fact that although 4 implants showed signs of mobility within the first year of the study, 2 of them quickly recovered and attained stability by the 2-year follow-up visit. This could be attributed to the bone remodeling process that was most prominent in the first year after implant insertion.

For implant success, soft tissue outcomes are as critical as marginal bone response and primary implant stability, especially in the anterior maxilla. Healthy soft tissue recovery at the implant site is not only important for implant function but also impacts esthetic outcomes and patient satisfaction [33, 34]. This study showed excellent soft-tissue outcomes with significant improvements in papilla index, plaque index, and PES between the time of implant insertion and the 5-year follow-up time point. Mombelli’s modified bleeding index also improved significantly from the time of definitive prosthesis delivery to the 3-year follow-up. This outcome is in general agreement with other studies [35–37] that have reported positive mid- and long-term soft-tissue outcomes for single implants placed in the anterior maxilla.

In an esthetically demanding area such as the anterior maxilla, patient satisfaction is paramount to the success of the implant. Only a limited number of studies, so far, have reported subjective patient-centered outcomes in addition to objective evaluations of esthetic assessment for implant-supported restorations, with some suggesting that the two do not correlate for implant placements in the esthetic zone [38–40]. In this study, patient satisfaction was assessed using the OHIP-14 questionnaire, which showed that the oral health status of patients significantly improved from the time of implant insertion to the 5-year follow-up. This result is comparable to the OHIP-14 evaluation from a 5-year prospective study with immediate provisionalization of implants replacing single missing teeth in the anterior maxilla [41]. Specifically, both studies report a strong and significant improvement maintained over time. Interestingly, in the published study, the improvement was more pronounced and more stable in patients, who received implants in healed sites as opposed to fresh extraction sockets. This observation is consistent with the results in the current study, and, furthermore, underscores the importance of a long-lasting functional and natural-looking restoration of single missing teeth in the maxillary esthetic zone for the well-being of a patient.

As per the study design, implants were placed either in healed sites (89.9%) or extraction sites that had healed for at least 8 weeks (10.1%), and all were immediately provisionalized. Despite the fact that healed sites represented the majority, the distribution of bone quantity was favorable, with most sites (94.9%) showing only a little or moderate resorption. Consequently, only 5 implant sites had received bone graft material prior to the start of the study, and concomitant bone grafting was performed during 16 implant placements. As expected, most implants (96%) were placed using a flap approach, reflecting the treating clinician’s choice to use the incision to recreate a naturally looking appearance of the soft tissue. The healthy peri-implant tissue response and its maintenance over 5 years reported in this study are likely to be at least in part associated with these favorable conditions but also emphasize the importance of clinical decision making, such as anticipating the soft tissue margin, performing bone augmentation where necessary, and careful planning to optimize implant position for best outcomes. Regarding immediate provisionalization, while most reports indicate that this procedure has similar esthetic results when compared to conventional loading protocols [42], it is paramount to recognize the technical challenge of this approach as well as the importance of the implant design. In this study, the implant had a tapered geometry, which enables implant placement between adjacent natural teeth [6], provides high primary stability [43–46], and improves esthetics by allowing a gradual expansion of the alveolar ridge [7]. Furthermore, the implant-abutment connection was conical, which is known to be tight and mechanically stable, and provides a built-in platform shift that has been shown to reduce inflammation and bone loss in peri-implant tissues [10] as well as to support favorable esthetic results [47].

Studies analyzing the long-term function of single-tooth implant-supported restorations are scarce in number [1, 48–50]. Of those, a recent meta-analysis has investigated the survival and complication rates of implant-supported single crowns in the maxillary esthetic zone, based on 29 published studies [11]. For implants with a conical connection (11 studies), this analysis reported an annual failure rate of 0.2%, which is slightly better than the 5-year CSR of 97.8% reported in the present study. By contrast, implant success reported in the meta-analysis would amount to a rate of 76% at 5 years for all studies independent of the connection type, which is significantly below the rate of 97.8% observed in this study. The mean marginal bone loss calculated in the meta-analysis was 0.6 mm, with a mean follow-up of 3 years, which is comparable to the 3-year bone loss of 0.74 mm reported in this study.

As outlined in the 1- and 3-year interim reports (17, 18), the main limitations to this study include inter-center variability, the varying reason behind the edentulism in different patients, as well as treatment decisions that were left to the treating clinician’s discretion. Because of this overall variability, it is impossible to attribute the good hard and soft tissue outcomes to one specific aspect of the treatment. Further investigations are needed to identify factors that strongly promote such long-term radiological and clinical outcomes. Another limitation of this study is that the esthetic assessment was based on Jemt’s papilla index and the PES, without other esthetic indices such as implant crown aesthetic index (ICAI), pink and white esthetic score (PES/WES), complex esthetic index (CEI), implant aesthetic score (IAS), subjective esthetic score [SES], or Rompen index.

With favorable cumulative survival and success rates of 97.8%, stable marginal bone after initial remodeling post-insertion, significant improvement in soft tissue outcomes, and patient oral related quality of life, the major clinical conclusion of this study is that immediately provisionalized tapered implants are a viable long-term treatment option for patients requiring single-tooth restorations in the maxillary esthetic zone. However, it should be highlighted that immediate implant provisionalization is technically challenging, requiring superior surgical and prosthetic skills compared to conventional loading, especially in the anterior maxillary region where esthetic outcomes are just as important as implant function.

## Conclusions

Within the limitations of this study, the results show that immediately provisionalized implants offer a valuable and reliable long-term treatment option that supports stable bone levels and favorable soft tissue response at implant insertion sites while providing patients with a highly satisfactory esthetic solution.
